# Prevalence and factors associated with thirst among postsurgical patients at University of Gondar comprehensive specialized hospital. Institution-based cross-sectional study

**DOI:** 10.1186/s41687-022-00476-5

**Published:** 2022-06-18

**Authors:** Kumlachew Geta Belete, Henos Enyew Ashagrie, Misganaw Mengie Workie, Seid Adem Ahmed

**Affiliations:** 1 Department of Anesthesia, College of Health Sciences, Debre Tabor University, Debre Tabor, Ethiopia; 2 Department of Anesthesia, College of Medicine and Health Sciences, University of Gondar, Gondar, Ethiopia

**Keywords:** Perioperative thirst, Post-operative care, Post-operative dehydration

## Abstract

**Introduction:**

Thirst is a powerfully distressing sensation that occurs most frequently in the immediate postoperative period. Postoperative thirst is prevalent, the moderate-to-severe type is estimated to affect 53.2–69.8% of patients and causes significant patient discomfort.

**Objective:**

The objective of this study was to assess the prevalence, and factors associated with postoperative thirst among surgical patients in PACU at the University of Gondar Comprehensive Specialized Hospital from April 20 to June 27, 2021.

**Methods:**

An institution-based cross-sectional study was conducted at the University of Gondar Comprehensive Specialized Hospital. A total of 424 participants were included in the study. Statistical analysis had performed using SPSS 26.00 version statistical software. Binary logistic regression analysis was performed to identify the association between the prevalence of postoperative thirst and independent variables and only variables with *p*-value < 0.2 were entered into the multivariable analysis. The strength of the association was presented by odds ratio and 95% Confidence interval. *P*-value < 0.05 was considered statistically significant.

**Result:**

The prevalence of postoperative thirst among postsurgical patients was 59% (95% CI = 54.74–64.13). Inadequate preloading (Adjusted odes ratio (AOR) = 2.137 95% CI 1.260–3.624), prolonged Nil Per Os (NPO) time (AOR = 13.80 95% CI 2.93–65.37), general anesthesia (AOR = 3.90 95% CI 3.56–11.25), and axillary body temperature ≥ 37.5 °C (AOR = 8.07 95% CI 3.63–17.96) were significantly associated with postoperative thirst. Low room temperature (< 20 °C) was protective for the occurrence of postoperative thirst (AOR = 0.162 95% CI 0.37–0.707).

**Conclusion and recommendations:**

The prevalence of postoperative thirst remains high and need commitment in close monitoring of PACU patients and immediate intervention. We also urge that high-level, ongoing research be conducted in this area, as postoperative thirst is a very common problem with a lot to discover.

## Introduction

Thirst is used as a marker for body homeostasis with regards to hydration, and if there are any changes, the body detects them and signals a need for water. The patient focuses on the extraordinary shift in well-being brought on by thirst, describing it as distressful discomfort proportional to its severity [1].

The perception of thirst and the desire to drink liquids to achieve satiety are conditioned physiological processes in all human beings. Surgical patients, especially, are even more thirsty due to prolonged preoperative fasting, medications used and perioperative fluid imbalance. These stimuli result in negative behaviors, such as stress, anxiety, irritability and despair, which intensify thirst discomfort in the perioperative period [2]. The prevalence of moderate to severe thirst ranges from 53.2 to 69.8% among postoperative patients and causes significant patient discomfort [3].

Although thirst is not normally considered an important issue during postoperative period, one must pay attention, considering its complexity and the fact that it is one of the most stressful and impetuous experiences in immediate postoperative period(IPP) [4].Thirst is a strong distressing sensation that suppresses all other sensations; patients complain about it often in the post-anesthesia care unit. Thirst affect sleep quality and increase anxiety in patients, which affects their life quality and thus raises concern from medical staff [5, 6].

If left untreated, thirst causes patients a great deal of discomfort [7]. It has substantial relationship with dry lips, dry mouth, thick tongue, dry throat, hypo salivation, desire to swallow, and the search for water in postoperative patients, and that result in postoperative patient distress [2]. The silence that permeates surgical patients reflects the undervaluation of thirst by health professionals. Perioperative thirst, even with high prevalence, is little questioned and treated. Silence is perhaps an indication of not forgetting a suffering that contributes to distressing hospital experiences [2].

The effect of preoperative fasting on thirst caused the most common discomfort, Patients were more disturbed about not being able to drink after midnight than by not being able to eat or sleep or by thinking about surgery [8]. If the human body is exposed to excessive thirst and it is not handled properly, it may result in a number of complications ranging from minor warning signs to life-threatening conditions such as heat injury, cerebral edema, seizures, hypovolemic shock, renal failure, coma, and death [9].

A cross-sectional study done in Brazil in 2015, found that preoperative fasting time causes postoperative thirst and, the intensity of thirst increases with the duration of NPO time (30). An RCT conducted in Iran in 2006 showed that preoperative adequate hydration (≥ 20 ml/kg bolus) can efficiently reduce the occurrence of postoperative thirst, and it was superior to low dose hydration [10].This study aims to assess the prevalence, and factors associated with postoperative thirst among surgical patients in post anesthesia care unit.

## Methods and materials

An institution-based cross-sectional study was conducted to determine the prevalence and factors associated with postoperative thirst among surgical patients at the University of Gondar Comprehensive Specialized Hospital; North West Ethiopia; April 20 to June 27, 2021.

The study included all adult patients who had undergone surgery at the University of Gondar Comprehensive Specialized Hospital and were admitted to PACU. Uncooperative patients, modified Ramsey sedation score of > 3, and known Pregnant patients were excluded. The ethical issue was approved and received ethical clearance by an ethical review committee of the college of medicine and health sciences at the University of Gondar Comprehensive Specialized Hospital. This paper is registered in research registry No 7213.

### Sample size determination and sampling technique

To determine the sample size, single population proportion formula was used. Since there was no similar previous study done similar with this topic in Ethiopia to guesses the key proportion to this study, so that we take a proportion of 50% by assuming a 95% of confidence interval with a 5% margin of error, and at 95% confidence level (P = 50% was taken). p = 0.5 and q = 1-p = 0.5, d = 5%, z_α/2_ = 1.96.

$${\text{n }} = \, \left( {{\text{Z}}\alpha /{2}} \right)^{{2}} {\text{p}}\left( {{1} - {\text{p}}} \right){\text{ n}} = \, \left( {{1}.{96}} \right)^{{2}} \times \, \left( {0.{5} \times 0.{5}} \right))/ \, \left( {0.0{5}} \right)^{{2}} = {384}.{16 } \approx { 385}$$

where n = sample size, P = Sample Proportion, q = 1-p d = desired a degree of precision, Z—the standard normal value at 95% confidence level.

The total calculated sample size was 424 after adding a 10% non-respondent rate. The data collection procedure included an observational checklist and interview-based questionnaire. The data collectors were instructed about how to select volunteer participants to observe and record their preoperative and intraoperative data.

### Study variables

The dependent variable was prevalence of postoperative thirst among post-surgical patients. Patients’ demographic variables, habits of life and procedure related variables like length of NPO time, amount of fluid intake, urgency of the procedure, type of the surgical procedure, type of anesthesia technique, drugs administered, duration of surgery, room temperature, the temperature of the patient, fluid balance of the patient was the independent variables included.

### Operational definitions

*Thirst* is defined as sensation and a symptom associated with a craving to drink, and a sensation of dryness in the mouth and throat [11].Verbal numerical scale (VNS), is a validated thirst measurement tool, which has verbal and numerical scales ranging from 0 to 10, with zero meaning no thirst and ten, the most intense thirst ever felt [2]. Then the prevalence of postoperative thirst among surgical patients, based on this assessment tool interpreted as a score of (0 to 3) was no clinically relevant thirst, 4 and above taken as clinically relevant thirst [4, 12].The modified Ramsey sedation score was used to determine the patients' capacity to answer questions after they had fully recovered consciousness.

*Body mass index (BMI)* a simple index of weight-for-height that is commonly used to classify overweight and obesity in adults. Subjects were categorised as underweight if BMI < 18.5 kg/m2, overweight if BMI was in the range 25.0–29.9 kg/m2, and obese if BMI ≥ 30.0 kg/m2 [13].

### Data collection instrument and procedure

Verbal numerical scale (VNS), is a validated thirst measurement tool, which has verbal and numerical scales ranging from 0 to 10, with zero meaning no thirst and ten, the most intense thirst ever felt, and it classifies the 0 to 3 as clinically relevant thirst and 4 and above as clinically non-relevant thirst, VNS permits to use the verbal description of the feeling of thirst [2]. Ethical clearance was obtained from Ethical Review committee of Medicine and Health Science, School of Medicine, University of Gondar. Written informed consent was obtained from each study participant after a clear explanation about the merits of the study. At the second postoperative hour (120 min) after the end of the surgery, patients’ level of consciousness cheeked with Ramsey score and if they can respond well, a VNS is administered to assess the prevalence of postoperative thirst by a trained data collector at Post anesthesia care unit.

### Data quality control

Before the beginning of the data collection process, data collector’s and data collection supervisors were given training; on how to manage the data collection process. Pre-testing was done at UOGCSH postoperative units, on 22 patients to check the questionnaire validity. Then the actual data collection was started by interviewing the patient and extracting data from the patient card.

### Data management and statistical analysis

Sociodemographic variables, the prevalence of postoperative thirst, and associated factors of thirst were entered into Epi data 4.3 version and transferred to SPSS version 26 statistical software for analysis. Descriptive statistics were conducted to summarize patient’s information and to determine the prevalence of postoperative thirst among surgical patients. To determine the association between the prevalence of thirst with potential predictors of thirst among postoperative surgical patients, the chi-square test was employed.

Binary logistic regression analysis had conducted to identify factors associated with postoperative thirst and bi-variable analysis were performed to determine each of the independent variables. Only variables with a *p*-value less than 0.2 during bi-variable analysis was entered into the multivariate analysis. We have used the Hosmer–Lemeshow test of goodness of fit to check the appropriateness of the analysis model. The strength of the association was presented by an adjusted odds ratio and 95% Confidence interval. Finally, data had been presented by using numbers or frequencies, percentages, figures, and tables. *P*-value < 0.05 were considered statistically significant.

## Results

### Sociodemographic characteristics of study participants

A total of 424 surgical patients above the age of 18 years were included in this study. More than half of the study participants (60.6%) were male, and the majority of them (72.6%) were between the ages of 18 and 40 years. More than three forth of the participants had normal BMI (70.2%) and the mean BMI of study participants was 20.7618 ± 2.84438. The majority of the participants (73.3%) were rural residents (Table 1).

**Table 1:** Socio-demographic characteristics of post-surgical patients at University of Gondar Comprehensive Specialized Hospital, Northwest Ethiopia, April 20 to June 27, 2021 (N-424)

Variables	Frequency	Percentage (%)
*Gender*		
Male	257	60.6
Female	167	39.4
*Residence*		
Rural	311	73.3
Urban	113	26.7
*Age (in years)*		
18–40	308	72.6
40–60	85	20.0
> 60	31	7.4
*BMI (kg/m*^*2*^*)*		
Underweight	85	20
Normal BMI	308	72.6
Overweight	31	7.4

#### Preoperative personal habits and health status of the patients

From the total of 424 participants, most of them 73 (17.2%) had a history of alcohol taking, 20 (4.7%) were smokers, and only 2 (0.5%) identified as having a history of khat chewing. Of the participants 43 (10.1%) having coexisting disease, of which was 13 (10.1%) were diabetes mellitus (DM), 20 (4.7%) hypertension(HTN), 8 (1.9%) heart disease, and 8 (1.9%) were other coexisting diseases. More than half (79.0%) of the participants were American society of anesthesiologist (ASA) physical status I (Table 2).From the total of 424 participants, most of them 73 (17.2%) had a history of alcohol taking, 20 (4.7%) were smokers, and only 2 (0.5%) identified as having a history of khat chewing. Of the participants 43 (10.1%) having coexisting disease, of which was 13 (10.1%) were diabetes mellitus (DM), 20 (4.7%) hypertension(HTN), 8 (1.9%) heart disease, and 8 (1.9%) were other coexisting diseases. More than half (79.0%) of the participants were American society of anesthesiologist (ASA) physical status I (Table 2).

**Table 2:** Preoperative personal habits and health status of postoperative surgical patients

Variables		Frequency	Percentage (%)
*Personal habits*			
Cigarette smoking	Yes	20	4.7
	No	404	95.3
Alcohol taking	Yes	73	17.2
	No	351	82.8
Khat chewing	Yes	2	0.5
	No	422	99.5
*Co-existing disease*			
DM	Yes	13	3.1
	No	411	96.9
HTN	Yes	20	4.7
	No	404	95.3
Heart disease	Yes	8	1.9
	No	416	98.1
Others	Yes	8	1.9
	No	416	98.1
*ASA status*			
ASA I		335	79.0
ASA II and above		89	21.0

#### Perioperative clinical and environmental factors

The mean and standard deviation of surgical duration was 110.15 ± 52.769 min. The median body temperature was 36.9 °C with an IQR of (36.3–37.3 °C). The average room temperature with standard deviation was (24.94 ± 2.289) with minimum and maximum values of 19 and 36.3 °C respectively (Table 3). The median absolute NPO time was 8.00 with an IQR of (7.00–10.00) with a minimum value of 1 h and a maximum value of 30 h. Preloading fluid was given for 384 (90.6%) of the participants, the mean of the preloading fluid amount was 14.36 ml/kg and SD of 9.07 and a minimum of 0 ml and a maximum of 60 ml/kg fluid was given. Out of this only 102 (24.1%) of the participants got an adequate amount of preloading fluid (≥ 20 ml/kg) (Table 3). seventy-eight (18.4%) of study participants underwent orthopedic surgery, 302 (71.2) general surgery, and 44 (10.4%) gynecologic surgery. In terms of anesthesia types, 424 patients 294 (69.3%) had general and 30 (30.7%) were operated under regional anesthesia. This study showed that 274 (64.6%) of the patient were taken atropine, 269 (63.4%) neostigmine. 17 (4.0%) of the patients administered one or more antihypertensive medications and 7 (1.07) patients were taking diuretics medications. Opioid were administered for 271 (63.9%) of the patients, which is 120 (28.3%) morphine, 104 (24.5%) tramadol, 76 (17.9%) fentanyl, 4 (9%) pethidine (Table 3).

**Table 3:** Perioperative environmental and clinical factors for postoperative surgical patients (N = 424)

Variables	Frequency	Percentage (%)
*Type of surgery*		
Orthopedic	78	18.4
General surgery	302	71.2
Gynecologic	44	10.4
*Type of anesthesia*		
General	294	69.3
Regional	130	30.7
*Surgical duration*		
Less than one hour	109	25.7
One to two hours	282	66.5
Above three hours	33	7.8
*NPO hour*		
Un extended NPO duration	396	93.4
Extended NPO duration	28	6.6
*Preload adequacy*		
Adequate preload 20 ml/kg	102	24.1
In adequate preload < 20 ml/kg	322	75.4
*Medications*		
Atropine (anticholinergic)	274	64.6
Neostigmine (anticholinesterase)	269	63.4
Opioid	271	63.9
Antihypertensive	17	4
Diuretics	7	1.7

### The prevalence of postoperative thirst

The overall prevalence of thirst in immediate postoperative patients was 59.4% (95% CI: 54.74–64.13) and the mean thirst score was (4.39 ± 2.976).

### Factors associated with postoperative thirst

Age of the patient, ASA class, type of surgical procedure, the urgency of the procedure, history of co-existing diseases, surgical duration, administration of atropine, and neostigmine were significant in the Bivariate analysis with the *p*-value ≤ 0.2, but not significant and fail to appear into the final multivariate logistic regression model. Five variables associate significantly with occurrence of thirst with the Hosmer–Lemeshow goodness-of ft test for logistic regression was not significant (*p* = 0.825) and omnibus significant test (*p* = 0.000) (Table4).

**Table 4:** Factors associated with prevalence of postoperative thirst at post anesthesia care unit

Variable	Thirst	No thirst	COR (95% CI)	AOR (95% CI)	*P*-value
*Age (in years)*					
18–40	189 (61.4%)	119 (38.6%)	1*	1	
40–60	49 (57.6%)	36 (42.4%)	1.929 (0.917–4.057)	2.066 (0.765–5.581)	0.152
Above 60	14 (45.2%)	17 (54.8%)	1.653 (0.722–3.782)	1.383 (0.481–3.975)	0.548
*Classification*					
I	193 (57.6%)	142 (42.4%)	1*	1	
II and above	59 (66.3%)	30 (33.7%)	1.447 (0.886–2.362)	1.997 (0.898–4.442)	0.90
*Urgency*					
Elective	90 (35.7%)	82 (47.7%)	1*	1	
Emergency	162 (64.3%)	90 (35.7%)	1.640 (1.105–2.434)	1.374 (0.809–2.335)	0.240
*NPO time class*					
< 15 h	227 (57.3%)	169 (12.7%)	1*	1	
≥ 15 h	25 (89.3%)	3 (10.7%)	6.204 (1.843–20.889)	13.80 (2.93–65.37)	0.001
*Fluid preloading*					
< 20 ml/kg	205 (63.7%)	117 (36.3%)	2.050 (1.306–3.218)	2.137 (1.26–3.62)	0.006
≥ 20 ml/kg	47 (46.1%)	55 (53.9%)	1*	1	
*Co-existing disease*					
Yes	31 (72.1%)	12 (27.9%)	1.870 (0.932–3.754)	1.625 (0.598–4.417)	0.342
No	221 (58.0%)	160 (42.0%)	1*	1	
*Anesthetic technique*					
General	198 (67.3%)	96 (32.7%)	2.903 (1.897–4.443)	3.90 (3.56–11.25)	0.007
Regional	54 (41.5%)	76 (58.5%)	1*	1	
*Atropine*					
Yes	186 (67.9%)	88 (32.1%)	2.690 (1.785–4.054)	1.613 (0.515–5.057)	0.412
No	66 (44.0%)	84 (56.0%)	1*	1	
*Neostigmine*					
Yes	182 (67.7%)	87 (32.3%)	2.540 (1.692–3.815)	0.594 (00.172–2.049)	0.410
No	70 (45.2%)	85 (54.8%)	1*	1	
*Type of surgery*					
Orthopedic	36 (46.2%)	42 (53.8%)	1*	1	
General	190 (62.9%)	112 (37.1%)	1.979 (1.197–3.272)	0.848 (0.411–1.750)	0.656
Gynecologic	26 (59.1%)	18 (40.9%)	1.685 (0.978–3.560)	1.512 (0.569–4.020)	0.408
*Surgical duration*					
< one hour	64 (58.7%)	45 (41.3%)	1*	1	
2–3 h	164 (58.2%)	118 (41.8%)	0.977 (0.624–1.531)	0.886 (0.506–1.551)	0.671
> 3 h	24 (72.7%)	9 (27.3%)	1.875 (0.797–4.412)	1.933 (0.674–5.542)	0.220
*Room temperature*					
Normal	17 (60.7%)	11 (39.3%)	1*	1	
Low	3 (20.0%)	12 (80.0%)	0.162 (0.37–0.707)	0.16 (0.37–0.70)	0.002
High	232 (60.9%)	149 (39.1%)	1.004 (0.459–2.211)	0.635 (0.25–1.61)	0.337
*Body temperature*					
Normal	113 (55.7%)	90 (44.3%)	1*	1	
Hypothermia	49 (40.2%)	73 (59.8%)	1.871 (1.186–2.950)	0.620 (0.373–1.030)	0.065
Hyperthermia	90 (90.9%)	9 (9.1%)	14.898 (6.864–32.33)	8.07 (3.63–17.96)	0.000

*COR* Crude odd ration, *AOR* Adjusted odd ratio, *CI* confidence interval

*Significant association from bivariate analysis, *p*-value < 0.05 taken as significant association from multivariate analysis

## Discussion

The perception of thirst and the desire to drink liquids to achieve satiety are conditioned physiological processes in all human beings. Surgical patients, especially, are even more thirsty for factors such as preoperative fasting and medications used. These stimuli result in negative behaviors, such as stress, anxiety, irritability and despair, which intensify thirst discomfort in the perioperative period [14].

This study aimed to investigate prevalence of postoperative thirst and the contributing factors associated with it. Our study found that thirst occurs in more than half of post-surgical patients (59.4%)).The result of this study is considerably high as compared to a studies done in Eritrea with prevalence of postoperative thirst (27.7%) [15] and Kenya (43.1%) [16]. This discrepancy might be underestimation of the problem and the quality of PACU differ from country to country. Moreover, most of the time clinicians don’t want to assess patients thirst. In the postoperative period, patients experience the most ruthless part of the thirst, after remaining fasting for excessively long periods, many times because of lack of knowledge of the surgical staff managing thirst in a safe manner in the perioperative period [17]. Even if we didn’t study the level of knowledge of Anesthetists and nurses they took course in their curriculum during their study. The ignorance of the problem and not giving emphasis is the main thing for high prevalence of thirst. On the other hand, the prevalence of postoperative thirst was low in comparison with studies conducted in Brazil (78.73%) in 2019 [2] and (89.9%) [4] in 2018. This difference could be attributed to a variation in the description of the problem and the techniques utilized to assess the prevalence of thirst.

A RCT study done in Iran conclude that inadequate fluid preloading was significantly associated with the prevalence of postoperative thirst, in line to this the present study showed inadequate fluid preloading was more likely to cause postoperative thirst when compared to adequately preloaded patients [10]. Studies showed that this may be due to the pre-loading effect of body hydration which suppresses the thirst center in the hypothalamus [18].

The results of our study also showed that preoperative NPO time was significantly associated with the prevalence of postoperative thirst. An absolute preoperative NPO time ≥ of 15 h increases the likely hood of postoperative thirst by 13.8 times than NPO time < 15 h). This finding is supported by a study from India, which shows NPO time greater than 15 h had a significant association with the increased prevalence of thirst [19]. The reason is that showed that the etiology of post-operative thirst is multifaceted and has not been yet completely elucidated. Among various pathophysiologic factors, preoperative fasting and perioperative fluid loss may lead to hyperosmolarity and hypovolemia [3]. Another study found that the length of fasting is associated with complications that affect the quality of the patients’ postoperative recovery and nurses’ work and should not permit the unnecessary extension of fasting [20]. This finding is supported by the fact that, as the duration of NPO increases, subjects’ thirst receptors activate more and produce higher thirst intensity scores [21] whereas the liquid diet enriched with carbohydrates in 2 h before surgical procedures may provide benefits such as decreased sensation of thirst. The reason for prolonged NPO times in our setup might be the number of schedule in a operation room is more than three patients and the anesthetists recommend NPO for all patient starting from the midnight, so that patient who wait for operation are at risk of prolonged NPO. And there is the institutionalized culture that advocates that fasting must be maintained at any cost in our set up. this culture is the same to the study done by Pierotti et al. [17]. Those findings demonstrate that the protocols of preoperative preparation are not performed, which can intensify the discomforts resulting from fasting—such as the thirst [22].

Our study showed that types of anesthesia had been found significantly associated with the prevalence of postoperative thirst. Patients who received general anesthesia have more likelihood of postoperative thirst than those who take regional anesthesia. Our finding was supported by studies done in India and Brazil [2, 19]. Our study was supported by another study done in Taiwan which stated that general anesthesia was an independent significant risk factor for the likelihood of postoperative thirst [3].literatures justify the association might be due to the effect of the various anesthetic drugs, intubation, and a prolonged NPO time for general anesthesia subjects [19]. These factors contribute to the occurrence of biochemical and hormonal reactions, thus triggering thirst [17]. Concerning the length of anesthesia and surgical procedure, there was no significant association between prevalence of thirst and length of the procedure, type of procedure as well as length of anesthesia.

The result of the present study also showed that body temperature had a significant association with the prevalence of postoperative thirst. Hyperthermia (> 37.5 °C) had more likely to cause thirst in postoperative patients than body temperature less than 37.5 °C. This could be due to explained by that hyperthermia increase metabolism which in turn increase thirst. This sensitizes neurons responsible for integrating temperature and water balance sensations [23].

The study done in Taiwan found that low room temperature was protective for the prevalence of thirst, similarly our study showed that low room temperature < 20 °C was a protective factor for the prevalence of thirst in comparison with room temperature ≥ 20 °C [3]. This could be due to a diminished thirst response in cold weather, which occurs when human blood vessels constrict to prevent blood from flowing easily to the extremities when the environment is cold pulling more blood to its core, the body can conserve heat [24].

## Conclusion

The prevalence of postoperative thirst is high in University of Gondar comprehensive specialized hospital. Body temperature > 37.5 °C, NPO time > 15 h, fluid preloading < 20 ml/kg, and general anesthesia associated significantly with prevalence postoperative thirst.

### Limitation of the study

The limitation of the present study was it includes only one institution and it was conducted at small sample size.

### Recommendations

We recommend keeping the patients’ body temperature in the normal range and preloading with adequate fluid (20 ml/kg). Furthermore, we advise against a prolonged NPO times. We also recommend to scholars to conduct high-level, multi-center ongoing research as postoperative thirst is a very common problem with a lot to discover.

## Data Availability

The data generated and analyzed will be available on a reasonable request from the corresponding author.

## References

[1] da Silva LCJR, Aroni P, Fonseca LF (2016). Tenho sede! Vivência do paciente cirúrgico no período perioperatório. Rev SOBECC.

[2] de Nascimento LA, Nakaya TG, Conchon MF, Garcia AKA, Pierotti I, Serato VM (2019). Prevalência, intensidade e desconforto da sede no paciente cirúrgico no pós-operatório imediato. Rev SOBECC.

[3] Lee CW, Liu ST, Cheng YJ, Chiu CT, Hsu YF, Chao A (2020). Prevalence, risk factors, and optimized management of moderate-to-severe thirst in the post-anesthesia care unit. Sci Rep.

[4] Pierotti I, Fracarolli IFL, Fonseca LF, Aroni P (2018). Evaluation of the intensity and discomfort of perioperative thirst. Esc Anna Nery.

[5] Conchon MF (2014). Perioperative thirst: an analysis from the perspective of the symptom management theory. Rev Esc Enferm USP.

[6] Xiaolan W (2018). The effect of nursing intervention of postoperative thirst in patients after laparoscopic cholecystectomy. Am J Nurs Sci.

[7] Nascimento LAD, Garcia AKA, Conchon MF, Aroni P, Pierotti I, Martins PR, Fonseca LF (2020). Advances in the management of perioperative patients' thirst. AORN J.

[8] Brosnan J, Nagy VT (1946). Perioperative thirst: a patient perspective. J Perianesth Nurs.

[9] Clinic M, Network N (2016) Dehydration can lead to serious complications. pp 1–2

[10] Chohedri AH, Matin M, Khosravi A (2006). The impact of operative fluids on the prevention of postoperative anesthetic complications in ambulatory surgery: high dose vs low dose. Middle East J Anesthesiol.

[11] Porth CM, Erickson M (1992). Physiology of thirst and drinking: implication for nursing practice. Heart Lung.

[12] Daniela L, Oliveira DL, Ribeiro C, De Farias L (2020) Evaluation of thirst dimensions 25(2):99–104

[13] World Health Organization (2000) Obesity: preventing and managing the global epidemic. Report of a WHO consultation. Vol 894, World Health Organization technical report series. Switzerland 11234459

[14] Alves L, Nakaya TG, Conchon MF, Korki A, Garcia A, Pierotti I (2019). Prevalence intensity and discomfort of thirst in surgical patients in the immediate post-operative period. Rev SOBECC.

[15] Andemeskel YM, Elsholz T, Gebreyohannes G, Tesfamariam EH (2020). Undesirable postoperative anesthesia outcomes at two national referral hospitals: a cross-sectional study in Eritrea. Anesthesiol Res Pract.

[16] Njoroge G, Kivuti-Bitok L, Kimani S (2017). Preoperative fasting among adult patients for elective surgery in a Kenyan referral hospital. Int Sch Res Not.

[17] Pierotti I, Korki A, Garcia A (2018). Avaliação do tempo de jejum e sede no paciente cirúrgico sed en el paciente quirúrgico. Rev Baiana Enferm.

[18] Bichet DG (2018). Vasopressin and the regulation of thirst. Ann Nutr Metab.

[19] Das N, Nursing MS, Principal V (2018). effect of a thirst bundle versus ice on thirst intensity and dryness of mouth among post operative patients in a selected hospital. Bengleru.

[20] de Pereira NC, Turrini RNT, de Poveda VB (2017). Perioperative fasting time among cancer patients submitted to gastrointestinal surgeries. Rev da Esc Enferm.

[21] Stotts NA, Arai SR, Cooper BA, Nelson JE, Puntillo KA (2015). Predictors of thirst in intensive care unit patients. J Pain Symptom Manag.

[22] Birolim MM (2020). Sede do paciente cirúrgico ortopédico no pós-operatório imediato sed del paciente quirúrgico ortopédico. Rev Baiana Enferm.

[23] Barney CC, Folkerts MM (1995). Thermal dehydration-induced thirst in rats: Role of body temperature. Am J Physiol Regul Integr Comp Physiol.

[24] Kenefick RW, Hazzard MP, Mahood NV, Castellani JW (2004). Thirst sensations and avp responses at rest and during exercise-cold exposure. Med Sci Sport Exerc.

